# CASBERT: BERT-based retrieval for compositely annotated biosimulation model entities

**DOI:** 10.3389/fbinf.2023.1107467

**Published:** 2023-02-14

**Authors:** Yuda Munarko, Anand Rampadarath, David P. Nickerson

**Affiliations:** ^1^ Auckland Bioengineering Institute, University of Auckland, Auckland, New Zealand; ^2^ The New Zealand Institute for Plant & Food Research Ltd., Auckland, New Zealand

**Keywords:** BERT, sentence-BERT, information retrieval, composite annotation embedding, RDF, ontology, biomodels, physiome model repository (PMR)

## Abstract

Maximising FAIRness of biosimulation models requires a comprehensive description of model entities such as reactions, variables, and components. The COmputational Modeling in BIology NEtwork (COMBINE) community encourages the use of Resource Description Framework with composite annotations that semantically involve ontologies to ensure completeness and accuracy. These annotations facilitate scientists to find models or detailed information to inform further reuse, such as model composition, reproduction, and curation. SPARQL has been recommended as a key standard to access semantic annotation with RDF, which helps get entities precisely. However, SPARQL is unsuitable for most repository users who explore biosimulation models freely without adequate knowledge of ontologies, RDF structure, and SPARQL syntax. We propose here a text-based information retrieval approach, CASBERT, that is easy to use and can present candidates of relevant entities from models across a repository’s contents. CASBERT adapts Bidirectional Encoder Representations from Transformers (BERT), where each composite annotation about an entity is converted into an entity embedding for subsequent storage in a list of entity embeddings. For entity lookup, a query is transformed to a query embedding and compared to the entity embeddings, and then the entities are displayed in order based on their similarity. The list structure makes it possible to implement CASBERT as an efficient search engine product, with inexpensive addition, modification, and insertion of entity embedding. To demonstrate and test CASBERT, we created a dataset for testing from the Physiome Model Repository and a static export of the BioModels database consisting of query-entities pairs. Measured using Mean Average Precision and Mean Reciprocal Rank, we found that our approach can perform better than the traditional bag-of-words method.

## 1 Introduction

In developing a biosimulation model, it is essential to provide a comprehensive description of the entities of the model related to processes, reactions, variables, mathematical equations and parameters. Formally, the COmputational Modeling in BIology NEtwork (COMBINE) community recommended the description in the form of semantic annotations using the Resource Description Framework (RDF) technology (Neal et al., 2019). With semantic annotations, complete and precise description is constructed in a composite manner involving various knowledge source terms and their relationships in a structured form (Gennari et al., 2011). Further, composite annotations are used as a community standard to encourage platform interoperability and sharing and collaboration between modellers (Gennari et al., 2021; Welsh et al., 2021). This work supports this recommendation and goes beyond the composite annotation structure to provide an entity retrieval method with a simple data structure that is easy to deploy. Moreover, the method should be general enough so it can be implemented for different domains with composite annotations.

This complete and precise description is beneficial for understanding the model and subsequently becomes the key to rediscovery for verification and possible reuse. Verification that includes model curation ensures experimental results’ validity, reproducibility and consistency. Scientists can then confidently compare models or evaluate their proposed approaches. More comprehensive usability will enable model composition, creating larger-scale models, which is an essential aspect of modelling human physiology as a whole (Bassingthwaighte, 2000) and understanding the human body (Hunter et al., 2002).

The standard technology to locate and manipulate data stored in RDF format is SPARQL Protocol and RDF Query Language (SPARQL). SPARQL is powerful for retrieving data specifically and precisely (Pérez et al., 2009), although it requires a rigid syntax query. This rigidity becomes a barrier for most users even if they already have enough knowledge regarding the RDF triple and ontologies to explore RDF. For expert users, their queries still may fail caused by misspellings, capitalisation, and ontology term variation. Therefore, text-based queries that can be composed freely, as in commercial search engines, are preferred, although the results are less precise.

The current state-of-the-art approaches offer a workaround by converting text-based queries to SPARQL by leveraging deep learning. Most of the works are for question-and-answer tasks on general knowledge, for example, about an object’s location and public figures’ achievements; hence, they do not explicitly support the search of entities annotated compositely. The knowledge base used is generic RDF triple graphs such as DBPedia^1^, Yet Another Great Ontology (YAGO)^2^, and Wikidata^3^. These graphs maintain a massive amount of information about entities and their facts, extracted from various sources such as Wikipedia and GeoName. The community and users manually validate information, so the level of accuracy is relatively high. Soru et al. (2020) and Yin et al. (2021) have considered the conversion as a language translation problem where SPARQL is the foreign language. Soru et al. (2020) implemented Long Short-Term Memory (LSTM) architecture to build sequence-to-sequence models and train the model over a dataset extracted from DBPedia. Then, Yin et al. (2021) extended the work by investigating the use of eight Neural Machine Translation (NMT) methods built using Convolutional Neural Network (CNN), Recurrent Neural Network (RNN), and Transformer. CNN-based method (Gehring et al., 2017) performed the best within these methods, followed by Transformer-based (Vaswani et al., 2017). However, in the context of natural language translation, the use of Transformer is prospective to improve performance since it is not as mature as RNN and CNN. With the popularity of Bidirectional Encoder Representations from Transformers (BERT) (Devlin et al., 2018), Tran et al. (2021) created SPBERT, a Transformer-based model pre-trained using a large DBPedia dataset for natural language to SPARQL and query results verbalisation tasks, and proved that the Transformer-based approach could surpass RNN and CNN. Adapting the created model for a new model is efficient by finetuning the existing model with less training data while keeping the property of the original model. Nevertheless, the text converted in these approaches must be in the natural language templates as formatted in the dataset. They cannot accommodate properly unstructured queries using keywords such as those used on commercial search engines.

Working well with structured and unstructured text-based queries, Natural Language Interface for Model Entity Discovery (NLIMED) provides an interface to retrieve entities annotated compositely (Munarko et al., 2022). It identifies phrases in the query associated with the physiological domain and links them to possible ontology classes and predicates. The link results are then composed as SPARQL and executed at the SPARQL endpoint to retrieve entities. A similar tool was developed by Sarwar et al. (2019), Model Annotation and Discovery (MAD), with the same domain but limited to entities in epithelial models. This limitation relates to template-based methods whose templates are customised for a particular topic, so adding topic coverage requires new templates.

This paper presents CASBERT, a method to retrieve entities in biosimulation models that are annotated compositely. We apply the information retrieval paradigm and leave the complexity of SPARQL providing a more expressive query composition while maximising the advantages of BERT. By adopting Sentence-BERT (Reimers and Gurevych, 2019), composite annotations describing entities are pre-calculated into entity embeddings and stored into a list of entity embeddings. With the entity embeddings, a query that is also converted into a query embedding is compared using a similarity formula; then, the ranked results are displayed.

With CASBERT, researchers can now explore information about entities in biosimulation models stored in a repository. They can find and access information quickly and adequately, including parameter values, variable units, variable types, and mathematical equations. CASBERT allows users to create expressive queries as keywords or natural language, such as ‘concentration of triose phosphate in astrocytes’. This convenience accelerates model validation, reproduction and reuse, directly supporting the principles of FAIR (Findability, Accessibility, Interoperability and Reusability) data.

Recently, the biosimulation model formats in the two largest repositories, the Physiome Model Repository (PMR) (Yu et al., 2011) and the BioModels Database (Chelliah et al., 2015), have used RDF to describe their entities. Composite annotations are largely used to describe models in CellML (Cuellar et al., 2003) and SBML (Hucka et al., 2003) formats, detailing entities with terms in ontologies such as anatomical location, chemical compound, physics of biology, gene, and protein. We generated test data from these repositories in query and entity pairs. CASBERT performance was measured using Mean Average Precision and Mean Reciprocal Rank, and compared to the traditional bag-of-words method, the score was significantly higher. In addition, entity embeddings stored as a list are easy to manage, allowing for efficient addition, subtraction, and replacement processes, making them suitable for search engine implementations. CASBERT can also be implemented for composite annotation search in various domains such as chemistry, pharmacy, and medicine. Our implementation, dataset, and experiment setup are publicly available^4^.

## 2 Materials and methods

CASBERT provides an approach to converting composite annotations defining entities and queries to embeddings. Entity embeddings are pre-calculated and stored in a list of entity embeddings, whereas query embeddings are created on the fly when a request is made. Entities are then presented from the most relevant by initially calculating the similarity values between query embedding and entity embeddings.

The conversion to embedding methods are based on Bidirectional Encoder Representations from Transformers (BERT) (Devlin et al., 2018) by implementing Sentence-BERT (Reimers and Gurevych, 2019). For the experiment, we created a dataset by collecting composite annotations from biosimulation models from the PMR and the BioModels database. These conversion methods are described in the following subsections, starting with the dataset used.

### 2.1 Biosimulation model - Composite Annotation Query (BM-CAQ) dataset

We constructed the dataset for the experiment by extracting compositely annotated entities in the PMR (Yu et al., 2011) and the BioModels database (Chelliah et al., 2015). The PMR is a repository storing biosimulation models initiated by the Physiome Project (Hunter et al., 2002). Most models are written using the CellML standard and annotated using the RDF standard. The models usually are equipped with human-readable information that can be loaded as web pages. We can further simulate and analyse physiological processes by running models using tools such as OpenCOR (Garny and Hunter, 2015). Like the PMR, the BioModels database manages biosimulation models in more significant numbers. The models deposited in this repository are mostly written using SBML standard (Hucka et al., 2003).

Table 1 presents the example entities described compositely using the RDF standard in the PMR. These entities are variables of the brain energy metabolism model (Cloutier et al., 2009) whose CellML model is available in the PMR^5^. An entity is described by at least one or, ideally, more ontology classes to provide detailed and precise descriptions. In the examples, *GAPg*/*GAPg* is annotated with OPB:00340, CHEBI:17138, and FMA:54537, whereas *dAMP*_*dATPn.ATPn* is annotated with OPB:00340, CHEBI: 15422, and FMA:54527, where these ontology classes are concepts available in Ontology of Physics for Biology (OPB) (Cook et al., 2008), Foundational Model of Anatomy (FMA) (Rosse and Mejino, 2008), and Chemical Entities of Biological Interest (ChEBI) (Degtyarenko et al., 2008), respectively. Additionally, the relationship between an entity and ontology classes is specified using predicates where in the example, there are ‘bqbiol:isPropertyOf’, ‘bqbiol:isPartOf’, ‘bqbiol:isVersionOf’ and ‘bqbiol:is’. As a complement, although not mandatory, an entity may be provided with a literal description marked with the ‘dcterms:description’ predicate, for example, ‘Rate of change in the concentration of glyceraldehyde-3-phosphate in the astrocyte’ on *GAPg*/*GAPg* entity.

**TABLE 1:** The example of compositely annotated entities written using the RDF standard in the PMR. These entities are variables of the brain energy metabolism model, GAPg/GAPg and dAMP_dATPn.ATPn (Cloutier et al., 2009). They are described by ontology classes such as OPB_00340, CHEBI:17138, and FMA:54527; and predicates such as ‘bqbiol:isVersionOf’ and ‘bqbiol:is’. There are also literal descriptions marked by the dcterms:description predicate, although not all entities have one.

Variable	Annotation using RDF
GAPg/GAPg	<rdf:Description rdf:about = “./cloutier_2009.cellml#**GAPg_GAPg**”>
	<bqbiol:isPropertyOf rdf:resource = “./cloutier_2009.cellml#entity_1”/>
	<**bqbiol:isVersionOf** rdf:resource = “ **https://identifiers.org/opb/OPB_00340** ”/>
	<**dcterms:description**>
	**Rate of change in the concentration of glyceraldehyde-3-phosphate in the astrocyte**
	</dcterms:description>
	</rdf:Description>
	<rdf:Description rdf:about = “./cloutier_2009.cellml#entity_1”>
	<bqbiol:isPartOf rdf:resource = “./cloutier_2009.cellml#entity_2”/>
	<**bqbiol:is** rdf:resource = “ **http://identifiers.org/chebi/CHEBI:17138** ”/>
	</rdf:Description>
	<rdf:Description rdf:about = “./cloutier_2009.cellml#entity_2”>
	<**bqbiol:is** rdf:resource = “ **http://identifiers.org/fma/FMA:54537** ”/>
	</rdf:Description>
dAMP_dATPn.ATPn	<rdf:Description rdf:about = “./cloutier_2009.cellml#**dAMP_dATPn.ATPn**”>
	<bqbiol:isPropertyOf rdf:resource = “./cloutier_2009.cellml#entity_15”/>
	<**bqbiol:isVersionOf** rdf:resource = “ **https://identifiers.org/opb/OPB_00340** ”/>
	<**dcterms:description**>
	**Rate of change in the concentration of adenosine triphosphate in the neuron of the brain**
	</dcterms:description>
	</rdf:Description>
	<rdf:Description rdf:about = “./cloutier_2009.cellml#entity_15”>
	<bqbiol:isPartOf rdf:resource = “./cloutier_2009.cellml#entity_1”/>
	<**bqbiol:is** rdf:resource = “ **http://identifiers.org/chebi/CHEBI:15422** ”/>
	</rdf:Description>
	<rdf:Description rdf:about = “./cloutier_2009.cellml#entity_1”>
	<**bqbiol:is** rdf:resource = “ **http://identifiers.org/fma/FMA:54527** ”/>
	</rdf:Description>

Text in bold are the main terms for the composite annotation, which are the ontology classes, variable description, and variable name whose relationship is described by the predicates.

There are 13,360 entities^6^ in the RDF and CellML files in the PMR, and 4,652 have literal descriptions. These literal descriptions usually accurately summarise the composite annotations of entities, although some are not. We assume that an accurate literal description can be used as a query representation for testing CASBERT and can reflect future user queries. Therefore, our test data includes all accurate literal descriptions as valid queries and the related entities as relevant search objects. Altogether, there are 338 unique queries, each relating to one or many entities. Then, we will refer to this set of query-entity pairs as *noPredicate* because most queries do not explicitly have an RDF predicate term.

However, we anticipate that queries for specific entity searches mimicking SPARQL will include predicate terms. Therefore, we created the second set of query-entity pairs by inserting the terms in the predicates to the queries in *noPredicate*. Terms were chosen randomly based on a series of predicates determining the relationship between ontology classes and entities. For example, the GAPg in Table 1 and CHEBI:17138 (glyceraldehyde-3-phosphate) are linked by ‘bqbiol:isPropertyOf’ and ‘bqbiol:is’ so that the insert can use the ‘is property of’ and ‘is’. If ‘is property of’ is selected, the new query becomes ‘Rate of change in the concentration of is property of glyceraldehyde-3-phosphate in the astrocyte’. We got 534 additional query-entity pairs and name this set as *withPredicate*’.

We applied the same strategy to extract queries from the BioModels database, which returned 834 *noPredicate* and 1,541 *withPredicate* queries. In its entirety, this dataset is named Biosimulation Model - Composite Annotation Query (BM-CAQ) and sample data are shown in Supplementary Tables S1–S4. The source code for creating this dataset and testing CASBERT is available online^7^.

### 2.2 Composite annotation search using BERT (CASBERT)

We used Sentence-BERT (Reimers and Gurevych, 2019), a BERT-based sentence encoder, to convert queries and composite annotations to embeddings and to classify the query to be compared to the appropriate entity embedding list. BERT provides pre-training models built based on massive corpora that can be fine-tuned with a smaller corpus to maximise performance for domain-specific uses (Devlin et al., 2018). A sentence is converted into embedding by splitting to tokens and then calculating each token’s embedding unique to the context, such as the surrounding tokens and position. Therefore, the same token in different sentences will have a different embedding. This attention to context correlates with high reliability in several natural language processing tasks, such as named entity recognition, concept extraction, and sentiment analysis, and is relatively better than non-context embedding (Arora et al., 2020; Taillé et al., 2020). In addition, the form of the token, which is part of the sentence, makes BERT more adaptive to typographical errors and variations of word writing.

The most straightforward approach to create a sentence embedding is by averaging the token embeddings; however, this often leads to poor performance (Reimers and Gurevych, 2019). Resolving this issue, Sentence-BERT offers a better concatenation method optimised for Semantic Textual Similarity (STS) by applying Siamese (Bromley et al., 1993) and Triplet loss (Weinberger and Saul, 2009) networks.

In the following, we describe the CASBERT mechanism for transforming composite annotations to entity embeddings and queries to query embeddings.

#### 2.2.1 Entity embedding

An entity embedding represents an entity as a dense vector whose dimensions match the sentence transformer model used. CASBERT generates this presentation by converting composite annotations to embedding. The annotation comprises triples which are subject, predicate and object expressions. These triples form a tree where the root is the entity’s name, the leaves are the ontology classes (objects), and the edges are the predicates. The predicates link the root and the leaves to form paths. The entity embedding is the average of all path embeddings, where the ontology class embedding and the predicate embeddings determine the path embedding.

**FIGURE 1:**
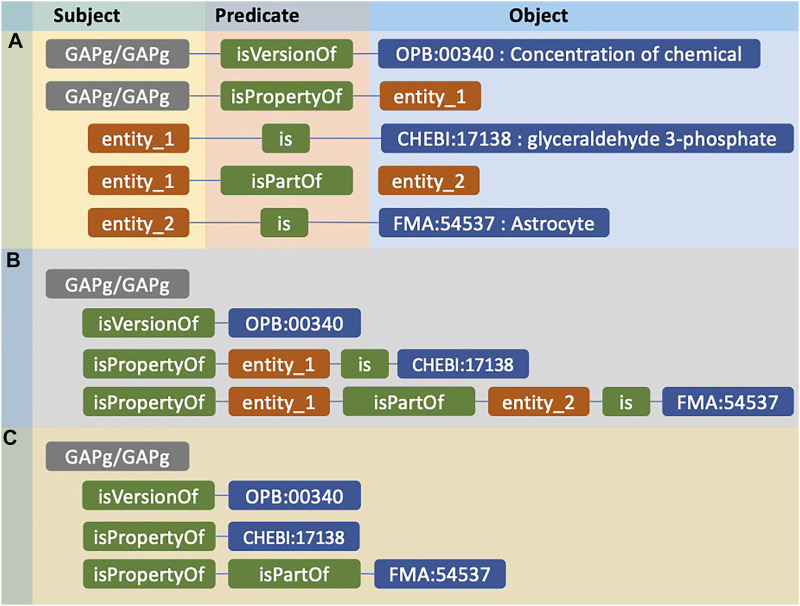
The example of an entity compositely annotated using RDF and its representation for further conversion to embedding. **(A)** The composite annotation of *GAPg*/*GAPg* entity by three ontology classes. **(B)** Paths consist of predicates connecting the entity to ontology classes. **(C)** The representation of the entity before converted to entity embedding.

Here we describe the process of converting composite annotations to entity embeddings. We use the *GAPg*/*GAPg* variable in Table 1 as a running example where all other composite annotations are processed similarly to become entity embeddings. Figure 1A shows the composite annotation regarding the concentration (OPB:00340) of glyceraldehyde 3-phosphate (CHEBI:17138) in an Astrocyte (FMA:54537). There are five triples with the subject and objects: root (*GAPg*/*GAPg*), ontology classes (OPB:00340, CHEBI:17138, FMA:54537), and intermediate subjects/objects (entity_1, entity_2). Figure 1B presents interconnected triples creating paths that display a clear relationship between root and ontology classes. The terms referred to as intermediate subject/object are usually generic and are similar across all composite annotations, so they cannot be used as a differentiator; therefore, we ignore them (Figure 1C). Next, we remove predicates that directly connect intermediate subjects, e.g. ‘is’, and ontology classes, because these only describe the intermediate subjects/objects, not the entity. We remove the prefix of the predicate term; for example, rather than ‘bqbiol:isPropertyOf’, we use ‘isPropertyOf’ as a predicate term.

**FIGURE 2:**
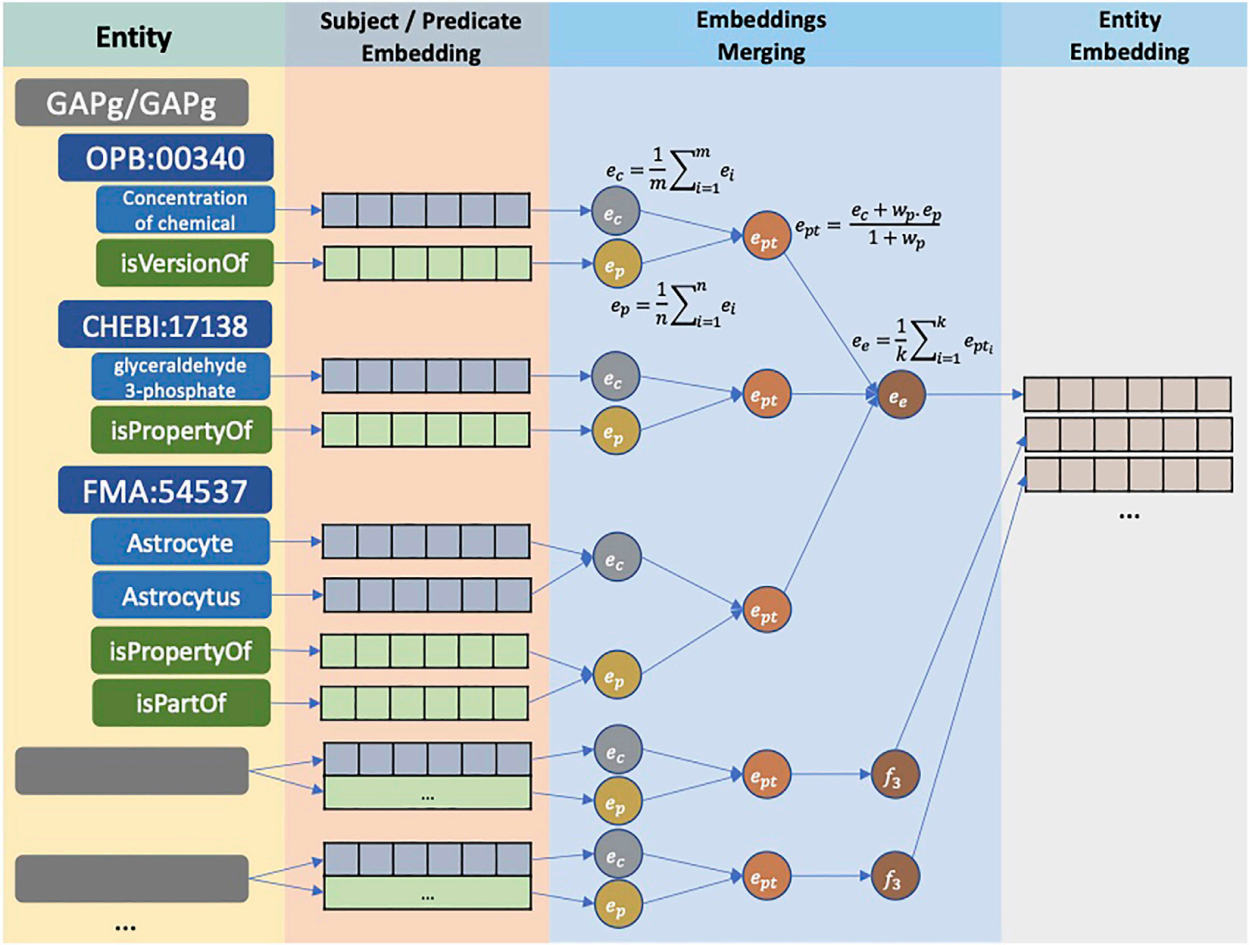
The conversion of entities to entity embeddings. For each entity, ontology classes and predicates are encoded into embeddings. Embeddings of ontology classes and predicates in one path are combined to form path embedding. Finally, all path embeddings are combined using the average function, creating an entity embedding.

Figure 2 illustrates the translation to the entity embedding process. Initially, CASBERT calculates the embedding of each path *e*_*pt*_ by combining its ontology class embedding *e*_*c*_ and the average of predicate embeddings *e*_*p*_ using Eq. 2; where *e*_*c*_ and *e*_*p*_ are calculated using Eq. (1). *e*_*c*_ is the average of ontology class feature embeddings; for example, FMA:54537 has a preferred label feature of ‘Astrocyte’ and a synonym feature of ‘Astrocytus’. There are other features, such as parent labels and definitions, but using the selected two features only can give a higher performance (Munarko et al., 2022). For *e*_*p*_ calculation, predicate terms in camelCase format are normalised to phrases in lowercase before converting to embeddings. For example, ‘isVersionOf’ and ‘isPartOf’ are changed to ‘is version of’ and ‘is part of’. Then, we limit the role of *e*_*p*_ with *w*_*p*_ between 0 and 1, which makes it lower than the role of the ontology class embedding. Finally, all path embeddings are averaged to get entity embedding *e*_*e*_ as presented by Eq. (3).

$$e_{c}=\frac{1}{m}\overset{m}{\underset{i=1}{\sum }}e_{i}\;and\;e_{p}=\frac{1}{n}\overset{n}{\underset{i=1}{\sum }}e_{i}$$ (1)

$$e_{pt}=\frac{e_{c}+w_{p}.e_{p}}{1+w_{p}}$$ (2)

$$e_{e}=\frac{1}{k}\overset{k}{\underset{i=1}{\sum }}{e_{pt}}_{i}$$ (3)

#### 2.2.2 Query embedding

Users create queries using keywords or natural language to search for entities. CASBERT represents these queries into query embeddings, so they are comparable to entity embeddings.

**FIGURE 3:**
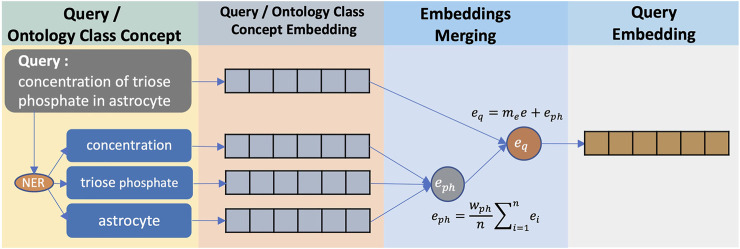
The conversion of a query to a query embedding. Phrases related to physiology and biochemical terms are identified using NER method, and then converted into embeddings using Sentence-BERT. These phrase embeddings are then averaged and combined with the entire query text embedding.

To explain the process of converting queries to query embeddings, we use ‘triose phosphate concentration in astrocytes’ as a running query example (see Figure 3). This query is intended to search for the *GAPg*/*GAPg* variable as in Table 1 where ‘triose phosphate’ is a synonym for ‘glyceraldehyde 3-phosphate’. A query can be assumed to be a composite annotation summary containing phrases about physiological and biochemical terms. CASBERT uses off-the-shelf natural language processing (NLP) method, SciSpacy (Neumann et al., 2019) with the ‘en_core_sci_scibert’ as Named Entity Recognition (NER) model, to identify these phrases; where for the query example, there are ‘concentration’, ‘triose phosphate’, and ‘astrocyte’. These phrases are converted to embeddings and combined by averaging (Eq. 4). We assume that these phrases correlate with ontology classes to some degree; therefore, the combined embedding *e*_*ph*_ is normalised by *w*_*ph*_, which is the average of the maximum similarity of each phrase in *p* to ontology classes *c*. After converting into embeddings, the similarity between *p*_*i*_ and *c*_*j*_ follows Eq. 6.

$$e_{ph}=\frac{w_{ph}}{n}\overset{n}{\underset{i=1}{\sum }}e_{i}\;where\;w_{ph}=\frac{1}{n}\overset{n}{\underset{i=1}{\sum }}\max\left(Sim_{j=1}^{m}\left(p_{i},c_{j}\right)\right)$$ (4)

$$e_{q}=m_{e}e+e_{ph}$$ (5)

$$Sim\left(e_{q},e_{e}\right)=\frac{e_{q}.e_{e}}{\left\|e_{q}\right\|\left\|e_{e}\right\|}=\frac{\sum_{i=1}^{n}e_{q_{i}}.e_{e_{i}}}{\sqrt{\sum_{i=1}^{n}e_{q_{i}}^{2}}\sqrt{\sum_{i=1}^{n}e_{e_{i}}^{2}}}$$ (6)

Furthermore, we take into account the relationship between phrases. This relationship is encoded explicitly or implicitly as conjunctions, prepositions, and the order of words. We capture this relationship by accounting for the overall query as an embedding *e*. The query embedding is then the addition of *e*_*ph*_ and *e*, where *e* is multiplied with the empirically decided multiplier *m*_*e*_ (see Eq. 5).

A few phrases may not have physiological or biochemical meaning and only complement other phrases. Therefore, their embeddings should be weighted lower and merged with the complemented phrase embedding. We use the *w*_*p*_ value for experiment purposes, the same weight used in Eq. (2), to weight these phrase embeddings.

#### 2.2.3 Entity retrieval

**FIGURE 4:**
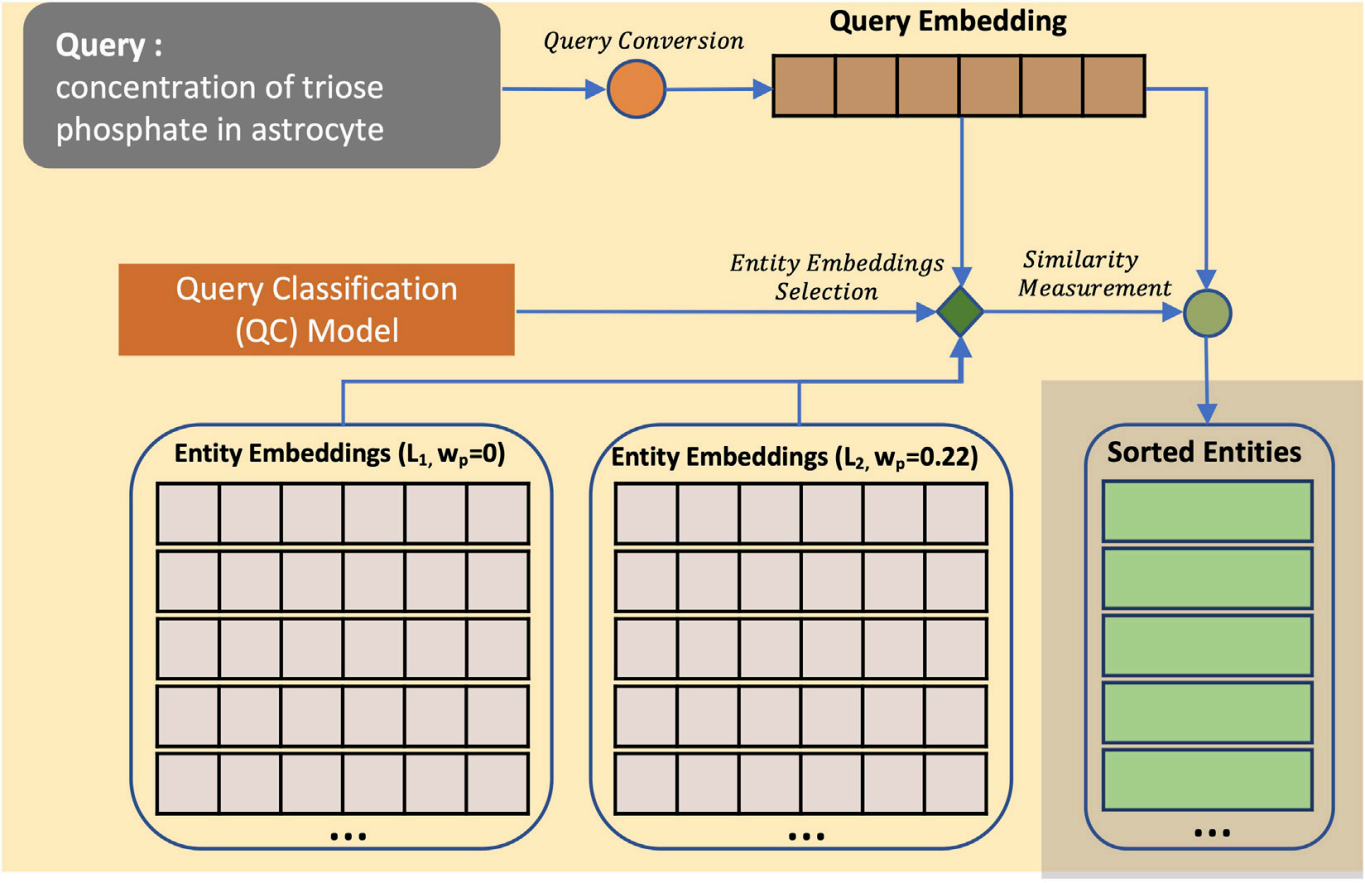
Entity search process using CASBERT. CASBERT provides entity embedding lists to choose from in the retrieval process according to the query type. Lists are distinguished by different *w*_*p*_ values, for example, *L*_1_ with a value of 0 and *L*_2_ with a value of 0.22. At first, the query is converted to an embedding. Then, the embedding becomes the input of the Query Classifier (QC) model to select the correct entity embedding list. The entity results are then taken from the chosen entity embedding list and presented in a sorted order based on cosine similarity values.

With the availability of the entity embedding list and query embedding, we can now calculate their similarities and present the relevant entities sorted from the most similar, as illustrated in Figure 4.

##### 2.2.3.1 Query - Entity Similarity

We implemented Cosine Similarity (CS) (Salton and McGill, 1983) to calculate the similarity value between a query embedding *e*_*q*_ and an entity embedding *e*_*e*_. CS of two embeddings is the dot product of both embeddings divided by the multiplication of the magnitude of both embeddings (Eq. (6)). Thus, CS ignores the magnitude of each embedding, making it suitable for the high dimensionality nature of embedding. Additionally, ‘multi-qa-MiniLM-L6-cos-v1’ pre-trained sentence transformer model (Reimers and Gurevych, 2019) used to convert a sentence to an embedding in CASBERT is optimised with CS. This calculated value is later used to display the retrieval results from the highest to the lowest.

##### 2.2.3.2 Query Classification

Considering that we can create multiple lists of entity embeddings with different *w*_*p*_ (see Eq. (2)), we found that some queries can retrieve best when compared to a list with *w*_*p*_ = 0, while others to a list with *w*_*p*_ > 0. As an experiment, we created two lists, *L*_1_ with *w*_*p*_ = 0 and *L*_2_ with *w*_*p*_ = 0.22. Then we created the Query Classification (QC) model to classify queries to *L*_1_ or *L*_2_, where these classes are associated with the embedding list used for entity retrieval. The training data was extracted from partially combined query-entity sets in BM-CAQ. We calculated the value of *mAP@*10 (see Eq. (8)) of the selected query pairs to perform searches to *L*_1_ and *L*_2_. The highest value determines pair labelling to *L*_1_ or *L*_2_. The QC model was trained using Transformers (Wolf et al., 2020) and the ‘bert-base-uncased’ pre-trained model where the training data was initially augmented using nlpaug^8^ to increase its size and diversity. The training process in more detail is presented in Supplementary Figure S1.

## 3 Experiments and results

### 3.1 Experiment setup

We conducted experiments to measure CASBERT’s performance in searching for entities in the biosimulation models in the PMR and the BioModels database. Table 2 displays query-entity pair sets and retrieval methods used in this experiment. The data used is the BM-CAQ dataset, including *noPredicate* and *withPredicate* sets. Additionally, we combined the two sets into *combine* and used 60% of it for training and validation of the QC model and the remaining 40% for additional test data.

**TABLE 2:** The strategies to measure CASBERT performance. There are three query sets and eight retrieval methods including BM25 as the gold standard.

Q-E type	# Of query-entities		Description
	PMR-CA	BioModels-CA	
*noPredicate*	338	834	The original query-entity pairs extracted from the PMR and the BioModels-CA.
*withPredicate*	534	1541	The expanded *noPredicate* set by randomly adding terms in composite annotation predicate to the associated existing query terms
*combine*	509	1777	Combination of *noPredicate* and *withPredicate* where the data used for QC model training is removed

Retrieval method	Terms used to generate query embedding		List of entity embeddings used
*macro*	whole query terms		*E*_1_(*w*_*p*_ = 0)
*macroWP*	whole query terms		*E*_2_(0 < *w*_*p*_ < 1)
*micro*	ontology class concept phrase		*E*_1_(*w*_*p*_ = 0)
*microWP*	ontology class concept phrase		*E*_2_(0 < *w*_*p*_ < 1)
*mixed*	whole query terms & ontology class concept phrases		*E*_1_(*w*_*p*_ = 0)
*mixedWP*	whole query terms & ontology class concept phrases		*E*_2_(0 < *w*_*p*_ < 1)
*mixedCl*	whole query terms & ontology class concept phrases		select between *L*_1_ or *L*_2_
BM25	Retrieval uses a bag-of-words method, BM25		

*w*_*p*_ is a variable that defines the role of the predicate embedding, which is ideally lower than the role of the ontology class embedding. In this experiment, we want to demonstrate the difference in performance between retrieval methods using entity lists without predicates and entity lists with predicates. Therefore, we first created two entity embedding lists, *L*_1_ with *w*_*p*_ = 0 and *L*_2_ with 0 < *w*_*p*_ < 1. The *w*_*p*_ value for *L*_2_ can be any arbitrary number as long as still in the correct range. Here, we choose 0.22 because it is small compared to the ontology class embedding weight, which is 1, and sufficient to represent the existence of predicates.

We tested three retrieval scenarios that yielded seven methods combined with entity embedding lists *L*_1_ and *L*_2_. We also measured the performance of BM25 (Robertson and Walker, 1994), a bag-of-words method, as the gold standard. The first scenario calculates the query embedding based on the query text only without identifying the phrases; when used to retrieve entities from *L*_1_ is named *macro* while from *L*_2_ is called *macroWP*. The following scenario uses phrases related to physiological and biochemical terms to generate query embeddings; when retrieving from *L*_1_, the method is called *micro*. The phrases are detected using the NER method, converted to embedding and combined using the averaging function. The additional use of non-physiological and biochemical phrases with this method, along with the retrieval from *L*_2_, is called *microWP*. Next, the third scenario combines the two initial scenarios using Eqss (4), (5), where the application for *L*_1_ and *L*_2_ are *mixed* and *mixedWP* consecutively. Here we set *m*_*e*_ = 1.9, which again is determined empirically by the logic that the query term as a whole is good enough to represent the query while the phrases within it can enhance the quality of the query representation as an embedding; therefore, *m*_*e*_ is more significant than *w*_*ph*_. Finally, we apply *L*_1_ and *L*_2_ selection using the QC model with the third scenario as *mixedCl*.

### 3.2 Evaluation metric

We measured CASBERT performance for each set of query-entity pairs *Q* in the BM-CAQ dataset using Mean Average Precision for the top k results (*mAP@k*), Eq. (8). *mAP@k* is based on Average Precision at k (*AP@k*) as shown by Equation 7, where *R* is the number of relevant entities in the results, *P@i* is the proportion of relevant entities in the top *i* results, and *r@i* is a relevance function that returns 0 or 1 for the irrelevance or relevance of the entity at position *i*, respectively. Suppose there is a query-entity pair (*q*, *es*) in *Q*, where the number of *es* is at least one; then, with the query *q*, a retrieval method should be able to retrieve entities in *es* only. The number of search results that match this *es* is *R*. Furthermore, we set the value of *k* to 10 because search results are usually arranged in pages of 10 entities, and users are generally only interested in the first page.

$$AP@k=\frac{1}{R}\overset{k}{\underset{i=1}{\sum }}P@i\times r@i$$ (7)

$$mAP@k=\frac{1}{\left|Q\right|}\overset{\left|Q\right|}{\underset{i=1}{\sum }}AP@k_{i}$$ (8)

Moreover, we also use Mean Reciprocal Rank (*mRR*) with Eq. (9) measuring the mean of the multiplicative inverse of the first entity in the retrieval results found to be relevant (*ranks*_*i*_). For example, given the query *q*, a retrieval method returns entities of *k* = 5 displayed in order of rank and relevance, 0 or 1, as {*1*: *0*,*2*: *1*,*3*: *0*,*4*: *0*,*5*: *1*}. Then the Reciprocal Rank calculation (*RR*) only considers the ranking of the first relevant entity, {*2*: *1*}, so the value of *RR* for *q* is 0.5. Then the value of *mRR* is calculated and averaged for all queries in *Q*.

$$mRR=\frac{1}{\left|Q\right|}\overset{\left|Q\right|}{\underset{i=1}{\sum }}\frac{1}{ranks_{i}}$$ (9)

### 3.3 Results

From Table 3, we can see that all of the methods used in CASBERT have higher *mAP@*10 and *mRR* than the gold standard BM25. The *macro* and *macroWP* perform reasonably well for all query-entity pair sets in the BM-CAQ. These methods are the most efficient because there is only one conversion to embedding for each query, so the retrieval process is faster. Meanwhile, the *micro* and *microWP*, which incorporate embeddings of phrases in the query, have the lowest measurement results among all the proposed methods. Using phrases alone overrides the relational information between phrases, resulting in lower performance. Strategies that combine embeddings of the whole query terms and phrases related to the concept of ontology classes (*mixed*, *mixedWP*, *mixedCl*) perform best (indicated by bold values in Table 3). These results show that the query text as a whole is sufficient to be converted into an embedding representing the query while embedding phrases helps increase the quality of query embedding. Moreover, in the *mixedWP*, using the QC model can slightly improve the retrieval quality and make it the best method. The QC model classifies queries for further retrieval from the appropriate entity embedding list, although the performance gains are modest.

**TABLE 3:** CASBERT performance over three query-entity pair sets and seven searching methods compared to the bag-of-words method (BM25) measured using *mAP@*10 and *mRR*. The numbers of entities in the PMR and the BioModels are 4,652 and 54,456 respectively.

Method	*noPredicate*		*withPredicate*		Combine	
	mAP@10	mRR	mAP@10	mRR	mAP@10	mRR
PMR-CA						
*macro*	0.645216	0.643424	0.572291	0.597649	0.601406	0.602604
*macroWP*	0.626565	0.624992	0.585014	0.606947	0.604012	0.608011
*micro*	0.619483	0.617890	0.534427	0.549304	0.592228	0.595516
*microWP*	0.606255	0.608560	0.504055	0.515426	0.574869	0.579472
*mixed*	0.656636	0.656326	0.572109	0.583003	0.623287	0.624638
*mixedWP*	0.641843	0.641940	0.585494	0.612151	0.615885	0.621419
*mixedCl*	**0.660346**	**0.659399**	**0.589960**	**0.615694**	**0.626420**	**0.631739**
BM25	0.459375	0.443034	0.464496	0.487373	0.456505	0.457420
BioModels-CA						
*macro*	0.345486	0.340106	0.310285	0.302129	0.356034	0.347905
*macroWP*	0.330100	0.326029	0.326831	0.325134	0.365903	0.362892
*micro*	0.294353	0.291914	0.283026	0.283291	0.312949	0.309471
*microWP*	0.301564	0.296179	0.274491	0.271451	0.314506	0.309211
*mixed*	0.347681	0.345045	0.326664	0.323393	0.370148	0.365433
*mixedWP*	0.335051	0.331222	0.**344296**	**0.344932**	0.379058	**0.377573**
*mixedCl*	**0.348054**	**0.344794**	0.343567	0.344058	**0.379351**	0.377557
BM25	0.240671	0.231515	0.294758	0.294779	0.280370	0.277879

The values in bold are maximum performance measured on three sets of query-entity pairs using mAP@10 and mRR values.

Based on the type of data for testing, the retrieval results from the PMR are better than those from the BioModels database. This difference is due to the more significant number of biosimulation models in the BioModels database, about twice the PMR, but their entities need to be annotated with more precision. By limiting the search to entities that are considered fully annotated, the performance is almost the same, as shown in the Supplementary Table S5.

**FIGURE 5:**
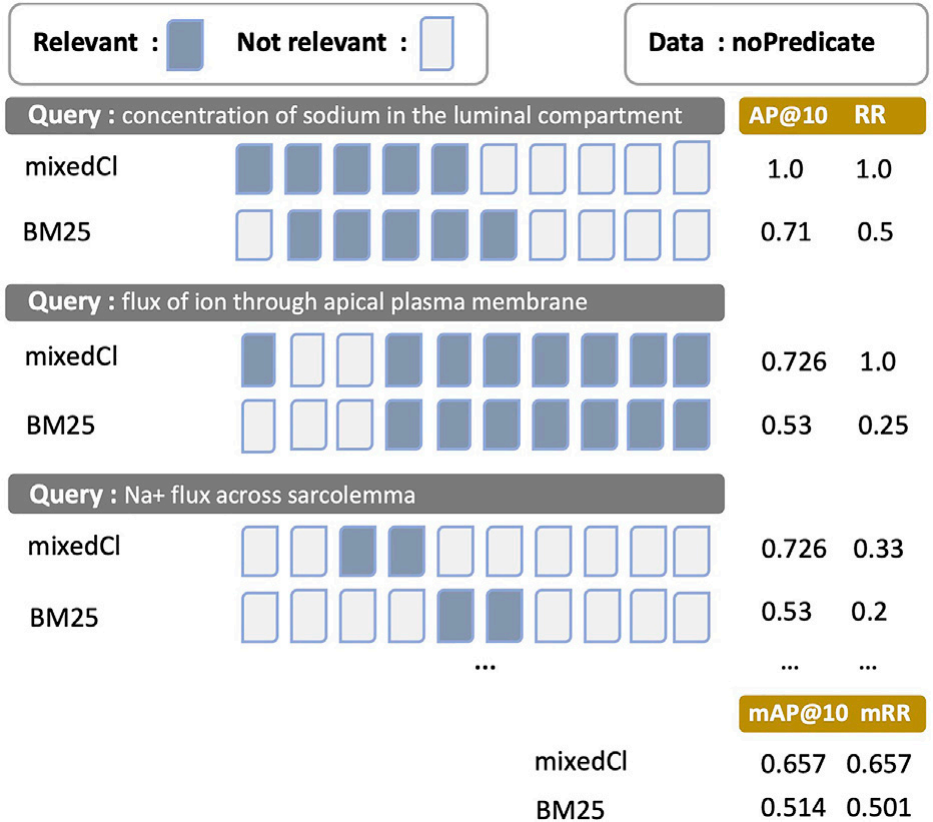
The presentation of search results differentiated by *mAP@*10 and *mRR* towards values. Blue boxes are relevant entities, while white boxes are irrelevant entities. *mAP@*10 and *mRR* are calculated from the results of the *mixedCl* and BM25 methods on the *noPredicate* query-entity pair set. Results are sorted in which entities with the higher similarity to the query are placed on the left-hand side. The results of the first and third queries are similar for both methods, but the use of *mixedCl*, with higher *mAP@*10 and *mRR*, can show relevant entities earlier than BM25.

Illustrating the effect of different *mAP@*10 and *mRR* values, Figure 5 shows the top 10 retrievals of entities for three query examples retrieved using *mixedCl* and BM25. Search results are organised in order from the left-hand side to the right-hand side based on the similarity values between queries and entity results, starting from the highest one. Three query-entity pairs are used in the *noPredicate* set. For the first and the third queries, *mixedCl* and BM25 retrieve the same number of relevant entities, indicated by blue boxes; however, the *mixedCl* presents the relevant entities earlier than BM25. Measured using *AP@*10 and *RR*, *mixedCl* raises higher values with a large margin. For the second example, *mixedCl* can retrieve more relevant entities and rank better. Overall, the *mAP@*10 and *mRR* of *mixedCl* is higher than BM25. A higher *mAP@*10 and *mRR* provide a better search experience with relevant entities served earlier or more.

## 4 Discussion

We have demonstrated that the adaptation of BERT-based embedding to encode entities’ composite annotations and queries in CASBERT can surpass the performance of the standard bag-of-words methods. This better performance is closely related to how BERT tokenises an input sentence and converts the token into embedding. BERT implements WordPiece tokeniser (Wu et al., 2016), a subword-based tokenisation algorithm. This algorithm can adequately accommodate wording variations, spelling errors, and missing white space issues. Therefore, unique words in specific domains, such as biology, can be segmented into appropriate tokens. These tokens are then converted into embeddings. For the token embedding to be unique to the position and sentence sequence, the representation of a token as an embedding is combined with the positional embedding and sentence sequence embedding. Hence, this combination can appropriately represent a sentence. Moreover, this way of embedding contributes to the generation of similar embeddings for sentences with synonymy meanings.

In the following subsections, we discuss CASBERT improvements, analysed based on different embedding generation methods. Then the discussion is followed by the possibility of implementing CASBERT in various domains and complementing SPARQL. Finally, we present our recommendations and future works.

### 4.1 Performance increase

As presented in Table 3, our proposed methods are quite effective in converting the entities’ composite annotations and queries to embeddings and retrieving relevant entities based on the provided query. The analysis of those methods and performance based on the similarity value between query and composite annotation is described below.

#### 4.1.1 Entity’s composite annotation to embedding methods

We have experimented with creating entity embedding by considering predicates (0 < *w*_*p*_ < 1) and not (*w*_*p*_ = 0). Entity embeddings considering predicates are primarily suitable for queries containing the predicate terms or their synonyms; conversely, the ones without predicates usually are suitable for queries without predicate terms. This pattern is shown by the consecutive good measurement results of the *mixedWP* method on *withPredicate* set and the *mixed* method on *noPredicate* set. Intuitively, for high-performance retrieval, the entity embedding list selected should match the presence of predicate terms in the query. However, this approach will not outperform the *mixedCl* method since the average results of the stated evidence are lower than the average results of the *mixedCl* method on the same sets. This lower performance may be because the terms identified as predicates are unrelated to either the predicate or the ontology class. On the other hand, predicates that are not phrases, but are conjunctions and prepositions, cannot be detected, eliminating the possibility of selecting the proper entity embedding list. The *mixedCl* method uses the QC model to determine the query embedding list based on the overall query terms. The application of a BERT-based classifier for classification is proven to have better accuracy. Overall, *mixedCl* is best on nine measurements of *mAP@*10 and *mRR* and only slightly lower than the best of the other three (Table 3, bold values).

While only two *w*_*p*_ values, 0 and 0.22, are used in this experiment, we predict that *w*_*p*_ should be adaptive to the query, so in the future, we recommend specifying *w*_*p*_ automatically. However, this adaptive *w*_*p*_ approach will sacrifice the simplicity of the current entity’s composite annotation list because ontology class and predicate embeddings should be separately managed, and there should be a mechanism to create entity embedding with given *w*_*p*_ value effectively.

#### 4.1.2 Query to embedding methods

The empirical results show that the mixed scenario performs best, followed by macro and micro scenarios consecutively. The micro scenario is intended to detect phrases related to physiology and biochemical terms in a query and generate a query embedding by combining all phrase embeddings. However, the detection accuracy depends on the NER method’s performance in identifying the phrases and the query created by the user.

The macro scenario is better than the micro scenario. This higher performance could be related to encoding whole query terms containing all phrases related to physiology and biochemical terms, including their relationship in a single embedding unit. However, individual phrases are not considered, allowing slight entity detection inaccuracies. Overall, this scenario is the most efficient because it only performs a one-step conversion from query to embedding, in contrast with the other scenarios that identify multiple phrases and convert to embeddings and then combine them.

The mixed scenario can slightly increase *mAP@*10 and *mRR*. As expected, this merge takes good account of the *macro* scenarios’s advantages and emphasises the critical phrases provided in the query. To avoid the decisive role of the phrases related to physiology and biochemical terms, delimiting with *w*_*ph*_ (Eq. 4) can give adequate proportion. Although this scenario is not the most efficient, its computational cost is linearly increased depending on the identified phrases. Due to its highest effectiveness, we recommend the mixed scenario to be implemented for composite annotation search.

#### 4.1.3 Performance analysis based on the similarity of query and Entity’s composite annotation

**FIGURE 6:**
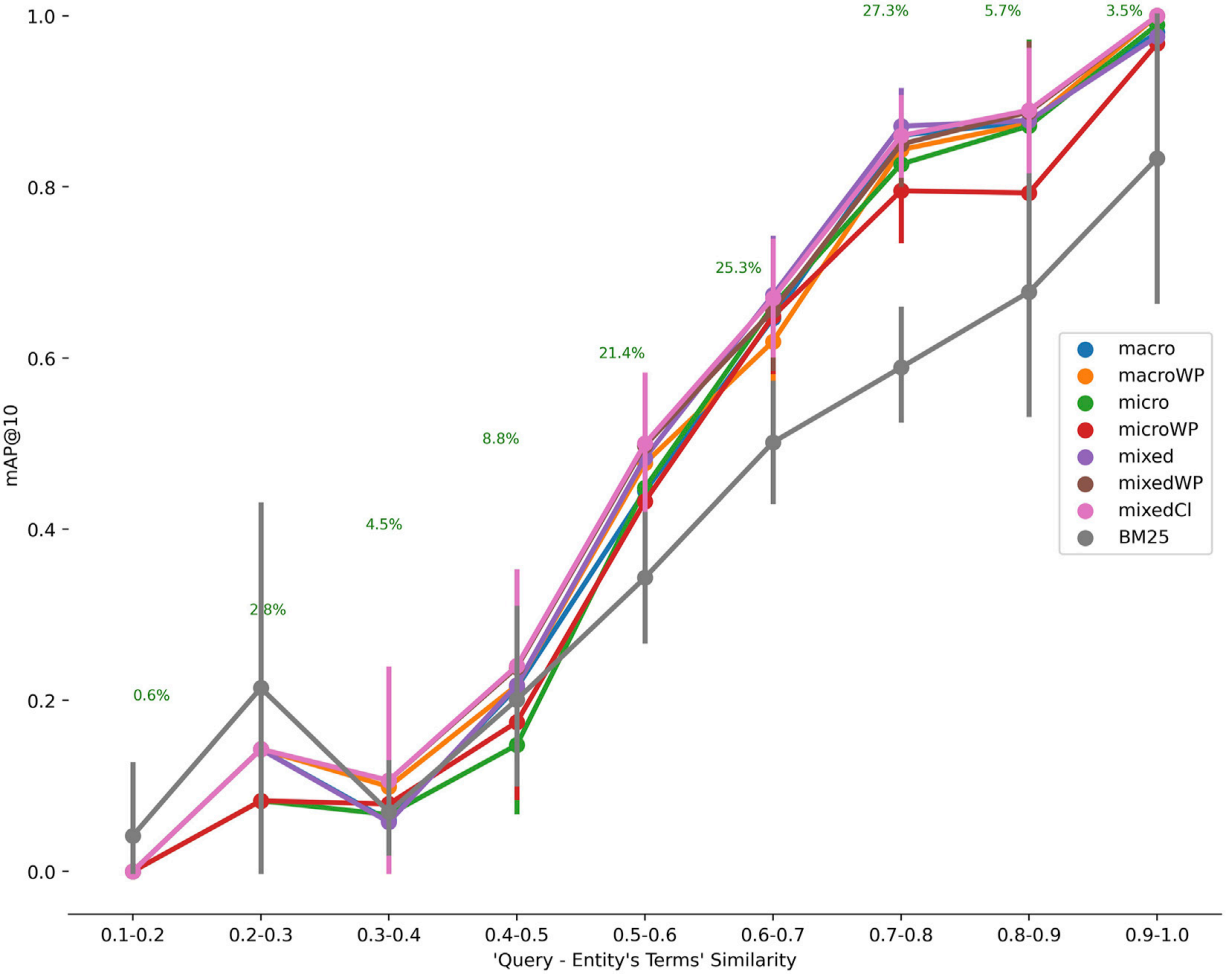
The relationship between the similarity of terms in the query with those in the entity to *mAP@*10 for PMR-CA. The number of entities is 4,652 and the number of test data is 509. Generally CASBERT is better than BM25 when the similarity value is 0.3 and above.

Figure 6 shows CASBERT’s ability in retrieving entities for various queries differentiated by their similarity to relevant entities for PMR-CA. We calculated the similarity directly using the query embedding generated with the *macro* method against the entity embedding. CASBERT performance is higher than BM25 when the similarity value is more than 0.3 and achieves the highest margin for similarity from 0.5 to 0.9, covering about 96% of the total test data. This pattern indicates CASBERT benefits because most user queries fall within this range. For very low query-entity similarity, 0.1 to 0.2, BM25 is better because a limited number of the same terms, one or two, can direct the query to relevant entities. In contrast, embedding in CASBERT may lead the query to entities with different terms but having the same context. Unfortunately, the combination of a low similarity value and the absence of a common term results in lower performance. Furthermore, we found a similar pattern for BioModels-CA, although we do not measure performance for low query-entity similarity values (see Supplementary Figure S2 and Supplementary Table S5).

### 4.2 Recommendations and future works

#### 4.2.1 Other domain implementation

We have shown CASBERT can well represent compositely annotated entities as embeddings. Although the data we use come from a repository of biosimulation models, implementation in other domains such as chemistry, pharmacy or medicine is possible as long as they are annotated using RDF and have ontology dictionaries. The entity retrieval process is started by modifying the input query to embedding using the *mixed* method or *macro* method for more straightforward implementation and then measuring query-entity similarity using cosine similarity. Moreover, most of the BERT models we used are pre-trained models without further fine tuning, except query classification modes; therefore, the implementation in other domains with no training data is still accessible.

#### 4.2.2 Search engine implementation

The embeddings representing entities now can be managed in a list of embeddings. The list is more straightforward than the standard indexing technique in search retrieval systems such as the inverted index. New embeddings can be easily attached to the list; even deletion, insertion, and replacement require only a minimum effort; therefore, overall maintenance will be cheaper. More importantly, we can avoid traditional search engine complexities, including preprocessing (stemming, case folding, stop word removal, spelling corrections, and lemmatisation), synonyms and abbreviations handlings.

#### 4.2.3 Cross repositories search

We estimate that it is possible to find similar information from different repositories with the same domain. Table 4 shows the example of two queries with their results from the PMR and the BioModels database. The query ‘concentration of triose phosphate in astrocyte’, ‘triose phosphate’ is correctly mapped to CHEBI:17138 in entities from both repositories, whereas ‘concentration’ and ‘astrocyte’ are mapped to different entities but with interrelated properties. Furthermore, the query ‘ammonium in cytoplasm’ also gives similar results with the mapping of ‘ammonium’ in entities from both queries was CHEBI:28938, while ‘cytoplasm’ as FMA:66836 (Portion of cytosol) in the PMR and GO:0005737 (cytoplasm) at the BioModels database. These results suggest that multiple repositories can be combined in a single search system to complement each other and be used for possible confirmation and reuse of models across repositories.

**TABLE 4:** The example of entities retrieved from different repositories, the PMR and the BioModels database. Those entities have similarities in the ontology classes related to the queries.

**Query**	**Result**	
	**PMR**	**BioModels database**
concentration of triose phosphate in astrocyte	**cloutier_2009.cellml#GAPg_GAPg**	**BIOMD0000000565.rdf#metaid_40**
	FMA:54537 Astrocyte	OPB:00592 Chemical molar flow rate
	OPB:00340 Concentration of chemical	CHEBI:32816 pyruvic acid
	CHEBI:17138 glyceraldehyde 3-phosphate	CHEBI:17138 glyceraldehyde 3-phosphate
		GO:0005829 cytosol
ammonium in cytoplasm	**weinstein_1995.cellml#Concentrations.C_int_NH4**	**BIOMD0000000470.rdf#metaid_1155**
	OPB:00340 Concentration of chemical	OPB:00592 Chemical molar flow rate
	CHEBI:28938 ammonium	CHEBI:28938 ammonium
	FMA:66836 Portion of cytosol	GO:0005576 extracellular region
		GO:0005737 cytoplasm

The text in bold are the variable names obtained from the PMR and the BioModel database using the two queries given.

#### 4.2.4 CASBERT for SPARQL

SPARQL and CASBERT have a similar intent to get information from RDF documents. SPARQL is rigid and can extract precise details as long as the information about the ontology and structure of the RDF document is known. In comparison, CASBERT is relaxed in exploring information freely where ontology knowledge is not mandatory. Therefore, both methods are not interchangeable, but CASBERT can supplement SPARQL to understand the structure of the RDF document and the ontology classes involved.

#### 4.2.5 Future works

We account for composite annotation structure and combine ontology class and predicate embeddings to calculate entity embedding. This combination involves two variables, *m*_*e*_ and *w*_*p*_, whose values adjust the search object and possibly the query type for maximum performance. We have currently prototyped a search engine^9^ that, once deployed, can collect query logs containing user activities in searching. Using these logs, we can analyse user behaviour and the relationship between the query and the relevant entity. We thought that by leveraging this relation and applying a grid search, CASBERT could automatically determine the value combinations of *m*_*e*_ and *w*_*p*_.

We are leveraging a Transformers-based method, Sentence-BERT, to convert text into embeddings. As an alternative, there is InferSent (Conneau et al., 2017), based on Bi-LSTM, and universal Sentence Encoder (USE) (Cer et al., 2018), based on Deep Averaging Network (DAN) and Transformers. Sentence-BERT is superior for sentiment analysis tasks but inferior to USE for TREC data for query classification tasks (Reimers and Gurevych, 2019). For our purposes, we believe Sentence-BERT is better than other methods because of its better understanding of context. The tokens used are WordPieces (Schuster and Nakajima, 2012) rather than words, so they can more precisely represent unique words in the biosimulation modelling domain and are more resistant to typos. In comparison, the method using Bi-LSTM combines the two-way conversion results, from left to right and *vice versa*, which are calculated separately. However, we will implement some text-to-embedding conversion methods as an option, as LSTM-based methods may be better for performance in other small data and systems with CPU only. Moreover, we will compare their performance for various purposes using CASBERT.

Further study in cross-repositories retrieval also needs to be considered; hence, it can promote reusability between repositories.

## 5 Conclusion

The increasing availability of composite annotation to describe entities in biosimulation models requires a simple tool to access information inside by ordinary users. We propose CASBERT, a BERT based method providing keyword-based searching that effectively manages composite annotations and retrieves entities using a text query. This effectiveness is achieved by converting the entities’ composite annotations to embeddings and organising them in a list; therefore, adding, deleting, inserting, and modifying embedding is cheaper. Getting relevant entities using a previously converted query to an embedding is straightforward with this structure. Using query-entities pairs test data extracted from the PMR and the BioModels database, empirically, CASBERT can retrieve better than bag-of-words methods such as BM25. It can potentially give a better user experience than the traditional approach. In the future, we are interested in developing a cross-repositories search engine to encourage biosimulation model reuse between different repositories.

## Data Availability

Publicly available datasets were analyzed in this study. This data can be found here: https://github.com/napakalas/casbert.

## Footnotes

1
https://www.dbpedia.org/.

2
https://yago-knowledge.org.

3
https://www.wikidata.org/.

4
https://github.com/napakalas/casbert/.

5
https://models.physiomeproject.org/workspace/5af/.

6 as of July 2020.

7
https://github.com/napakalas/casbert-experiment/.

8
https://github.com/makcedward/nlpaug.

9
https://github.com/napakalas/bmse/.
