# Supplementary material for: CASBERT: BERT-based retrieval for compositely annotated biosimulation model entities
Source: Front Bioinform. 2023 Feb 14;3:1107467. doi: 10.3389/fbinf.2023.1107467 (PMC9971925; doi:10.3389/fbinf.2023.1107467)
Supplement: Supplementary file 1 [file DataSheet1.PDF]

Supplementary Material

This document is supplementary material for CASBERT: Composite Annotation Search Based on BERT. Source code, dataset, and experimental setup are available at https://github.com/napakalas/casbert.

1 BIOSIMULATION MODEL - COMPOSITE ANNOTATION QUERY (BM-CAQ)

Table S1. Example of query-variables data in the query set in the biosimulation models in the PMR. This set contains a list of queries, the variables associated with the query, and the highest similarity values between the query and the variable variables.

Query	Variable ID	Max Score
Calcium-activated potassium current	’VarId-26893’, ’VarId-19743’	0.733406484
Concentration of sodium in the part of cytosol	’VarId-19766’, ’VarId-26916’	0.804430664
Calcium concentration in bulk cytosol	’VarId-112787’, ’VarId-112086’, ’VarId-64043’	0.726958036
Volume of the cytosolic fluid in the diadic space	’VarId-112102’, ’VarId-112104’, ’VarId-112782’	0.715647697
Total cytosolic volume	’VarId-117177’, ’VarId-112102’, ’VarId-112104’, ’VarId-112782’	0.723076522

Table S2: An example of variables in the PMR annotated with an ontology class with its predicates.

Variable ID	Class ID	Class Name	Predicate
VarId-26893	GO:0015269	calcium-activated potassium channel activity	’isComputationalComponentFor’, ’physicalPropertyOf’, ’hasMediatorParticipant’, ’hasPhysicalEntityReference’, ’hasPhysicalDefinition’
	OPB:00318	Charge flow rate	’isComputationalComponentFor’, ’hasPhysicalDefinition’
VarId-19743	GO:0015269	calcium-activated potassium channel activity	’isPropertyOf’, ’hasMediatorParticipant’, ’hasPhysicalEntityReference’
	OPB:00318	Charge flow rate	’isVersionOf’
VarId-19766	CHEBI:29101	sodium(1+)	’isPropertyOf’
	FMA:226054	Cytosol of neuron	’isPropertyOf’, ’isPartOf’
	OPB:00340	Concentration of chemical	’isVersionOf’
VarId-26916	CHEBI:29101	sodium(1+)	’isComputationalComponentFor’, ’physicalPropertyOf’, ’hasPhysicalDefinition’
	FMA:226054	Cytosol of neuron	’isComputationalComponentFor’, ’physicalPropertyOf’, ’part_of’, ’hasPhysicalDefinition’
	OPB:00340	Concentration of chemical	’isComputationalComponentFor’, ’hasPhysicalDefinition’
VarId-112782	FMA:66836	Portion of cytosol	’isPropertyOf’, ’is’
	FMA:14067	Cardiac myocyte	’isPropertyOf’, ’isPartOf’, ’is’
	OPB:00154	Fluid volume	’isVersionOf’
	OPB:00340	Concentration of chemical	’isVersionOf’

Continued on next page

VarId-112086

Table S2 – *Continued from previous page*

Variable ID	Class ID	Class Name	Predicate
	FMA:66836	Portion of cytosol	'isPropertyOf', 'isPartOf'
	FMA:14067	Cardiac myocyte	'isPropertyOf', 'isPartOf', 'isPartOf', 'is'
	CHEBI:29108	calcium(2+)	'isPropertyOf'
VarId-64043	OPB:00340	Concentration of chemical	'isVersionOf'
	FMA:66836	Portion of cytosol	'isPropertyOf', 'isPartOf'
	FMA:14067	Cardiac myocyte	'isPropertyOf', 'isPartOf', 'isPartOf', 'is'
	CHEBI:29108	calcium(2+)	'isPropertyOf', 'is'
VarId-112102	FMA:66836	Portion of cytosol	'isPropertyOf', 'is'
	FMA:14067	Cardiac myocyte	'isPropertyOf', 'isPartOf', 'is'
	OPB:00154	Fluid volume	'isVersionOf'
VarId-112104	FMA:14067	Cardiac myocyte	'isPropertyOf', 'isPartOf', 'isPartOf', 'is'
	FMA:66836	Portion of cytosol	'isPropertyOf'
	OPB:00154	Fluid volume	'isVersionOf'
VarId-112787	OPB:00340	Concentration of chemical	'isVersionOf'
	FMA:66836	Portion of cytosol	'isPropertyOf', 'isPartOf', 'is'
	CHEBI:29108	calcium(2+)	'isPropertyOf'
	FMA:14067	Cardiac myocyte	'isPropertyOf', 'isPartOf', 'isPartOf', 'is'
VarId-117177	OPB:00154	Fluid volume	'isVersionOf'
	FMA:66836	Portion of cytosol	'isPropertyOf'

Table S3. Example of query-variables data in the query set in the biosimulation models in the BioModels database. This set contains a list of queries, the variables associated with the query, and the highest similarity values between the query and the variable variables.

Query	Variable ID	Max Score
Extracellular glucose kinetics	'BIOMD0000000051.rdf#metaid_68', 'BIOMD0000000061.rdf#metaid_27', 'BIOMD0000000565.rdf#metaid_75'	0.720707416
squalene epoxidase (NADP)	'BIOMD0000000471.rdf#metaid_908', 'BIOMD0000000473.rdf#metaid_972', 'BIOMD0000000472.rdf#metaid_909', 'BIOMD0000000472.rdf#metaid_908', 'BIOMD0000000496.rdf#metaid_4246', 'BIOMD0000000497.rdf#metaid_4262', 'BIOMD0000000471.rdf#metaid_909'	0.729963005
Factor Xa lipid binding	'BIOMD0000000332.rdf#metaid_90', 'BIOMD0000000334.rdf#metaid_86', 'BIOMD0000000333.rdf#metaid_62'	0.709653497

Table S4: An example of variables in BioModels database annotated with an ontology class with its predicates.

Variable ID	Class ID	Class Name	Predicate
BIOMD0000000051.rdf#metaid_68	OPB_00592	Chemical amount flow rate	'isVersionOf'
	CHEBI:4167	D-glucopyranose	'isPropertyOf', 'hasSinkParticipant', 'hasPhysicalEntityReference'
	GO:0005576	extracellular region	'isPropertyOf', 'hasSinkParticipant', 'hasPhysicalEntityReference', 'isPartOf'
BIOMD0000000061.rdf#metaid_27	OPB_00592	Chemical amount flow rate	'isVersionOf'
	GO:0015758	glucose transmembrane transport	'isPropertyOf', 'isVersionOf'
	CHEBI:17234	glucose	'isPropertyOf', 'hasSourceParticipant', 'hasPhysicalEntityReference'
	GO:0005576	extracellular region	'isPropertyOf', 'hasSourceParticipant', 'hasPhysicalEntityReference', 'isPartOf', 'isVersionOf'
	GO:0005829	cytosol	'isPropertyOf', 'hasSourceParticipant', 'hasPhysicalEntityReference', 'isPartOf', 'isVersionOf'
BIOMD0000000565.rdf#metaid_75	OPB_00592	Chemical amount flow rate	'isVersionOf'
	CHEBI:17234	glucose	'isPropertyOf', 'hasSinkParticipant', 'hasPhysicalEntityReference', 'isVersionOf'
	GO:0005576	extracellular region	'isPropertyOf', 'hasSinkParticipant', 'hasPhysicalEntityReference', 'isPartOf', 'isVersionOf'
BIOMD0000000471.rdf#metaid_908	OPB_00592	Chemical amount flow rate	'isVersionOf'
	CHEBI:57945	NADH(2-)	'isPropertyOf', 'hasSourceParticipant', 'hasPhysicalEntityReference'
	CHEBI:15441	(S)-2,3-epoxysqualene	'isPropertyOf', 'hasSinkParticipant', 'hasPhysicalEntityReference'

Continued on next page

Table S4 – *Continued from previous page*

Variable ID	Class ID	Class Name	Predicate
	P32476	Squalene monooxygenase	'isPropertyOf', 'hasMediatorParticipant', 'hasPhysicalEntityReference'
	CHEBI:57540	NAD(1-)	'isPropertyOf', 'hasMediatorParticipant', 'hasPhysicalEntityReference'
	CHEBI:15379	dioxygen	'isPropertyOf', 'hasSourceParticipant', 'hasPhysicalEntityReference'
	CHEBI:15440	squalene	'isPropertyOf', 'hasSourceParticipant', 'hasPhysicalEntityReference'
	GO:0005737	cytoplasm	'isPropertyOf', 'hasSourceParticipant', 'hasPhysicalEntityReference', 'isPartOf'
	GO:0005576	extracellular region	'isPropertyOf', 'hasSourceParticipant', 'hasPhysicalEntityReference', 'isPartOf'
BIOMD0000000473.rdf#metaid_972	OPB_00592	Chemical amount flow rate	'isVersionOf'
	CHEBI:15441	(S)-2,3-epoxysqualene	'isPropertyOf', 'hasMediatorParticipant', 'hasPhysicalEntityReference'
	CHEBI:15379	dioxygen	'isPropertyOf', 'hasMediatorParticipant', 'hasPhysicalEntityReference'
	CHEBI:15440	squalene	'isPropertyOf', 'hasSourceParticipant', 'hasPhysicalEntityReference'
	CHEBI:57945	NADH(2-)	'isPropertyOf', 'hasSourceParticipant', 'hasPhysicalEntityReference'
	CHEBI:57540	NAD(1-)	'isPropertyOf', 'hasMediatorParticipant', 'hasPhysicalEntityReference'
	P32476	Squalene monooxygenase	'isPropertyOf', 'hasMediatorParticipant', 'hasPhysicalEntityReference'
	GO:0005737	cytoplasm	'isPropertyOf', 'hasMediatorParticipant', 'hasPhysicalEntityReference', 'isPartOf'
	GO:0005576	extracellular region	'isPropertyOf', 'hasMediatorParticipant', 'hasPhysicalEntityReference', 'isPartOf'

2 QUERY CLASSIFIER TRAINING

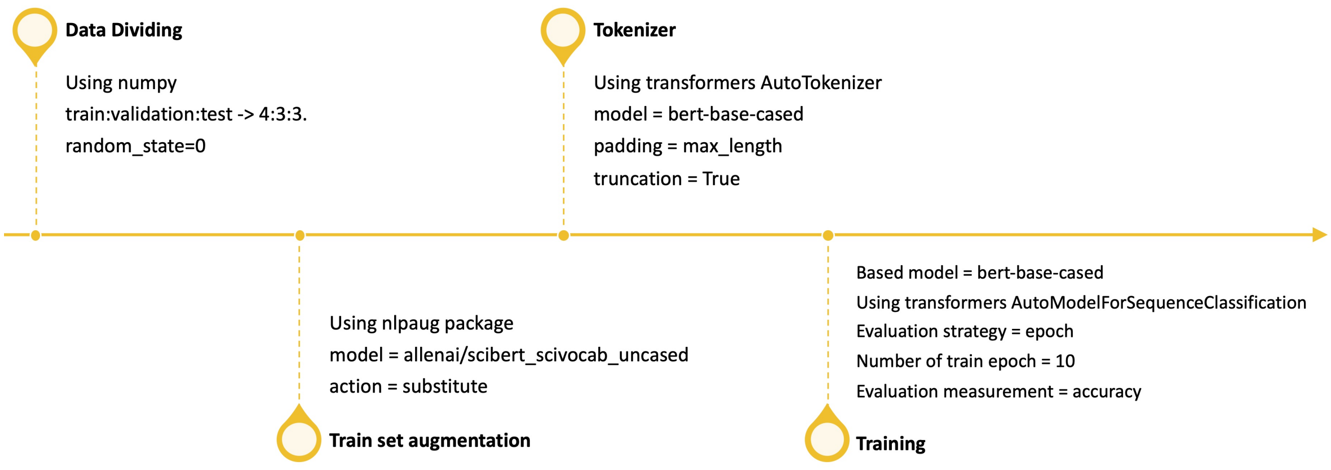

Figure S1: Setup for training Query Classifier (QC) model. At first, the dataset is divided into the train, validation, and test sets. The train set is augmented with nlpaug due to the limited number and then tokenised. The number of tokens per query is adjusted to the maximum of tokens used by the BERT model. The training was conducted with ten epoch strategies and evaluated based on accuracy.

3 CASBERT PERFORMANCE FOR BIOMODELS-CA

Table S5. CASBERT performance over Biomodels-CA and seven searching strategies compared to the bag-of-words method (BM25) measured using mAP@10 and mRR. The entities used are only 1,538, which are in BioModels-CA.

**Method**	***noPredicate***		***withPredicate***		**combine**	
	**mAP@10**	**mRR**	**mAP@10**	**mRR**	**mAP@10**	**mRR**
**BioModels-CA**						
macro	0.664723	0.654946	0.601524	0.601969	0.663477	0.659024
macroWP	0.625817	0.617877	0.594065	0.600354	0.652178	0.651609
micro	0.609719	0.607580	0.573874	0.575732	0.616996	0.614432
microWP	0.600927	0.600581	0.528423	0.531402	0.591117	0.589172
mixed	0.667825	0.659259	0.618766	0.621912	0.675504	0.673826
mixedWP	0.634301	0.628718	0.609025	0.618017	0.664714	0.665537
mixedCl	**0.669317**	**0.662252**	**0.630671**	**0.636152**	**0.678711**	**0.678250**
BM25	0.545968	0.537574	0.535652	0.541396	0.563251	0.561383

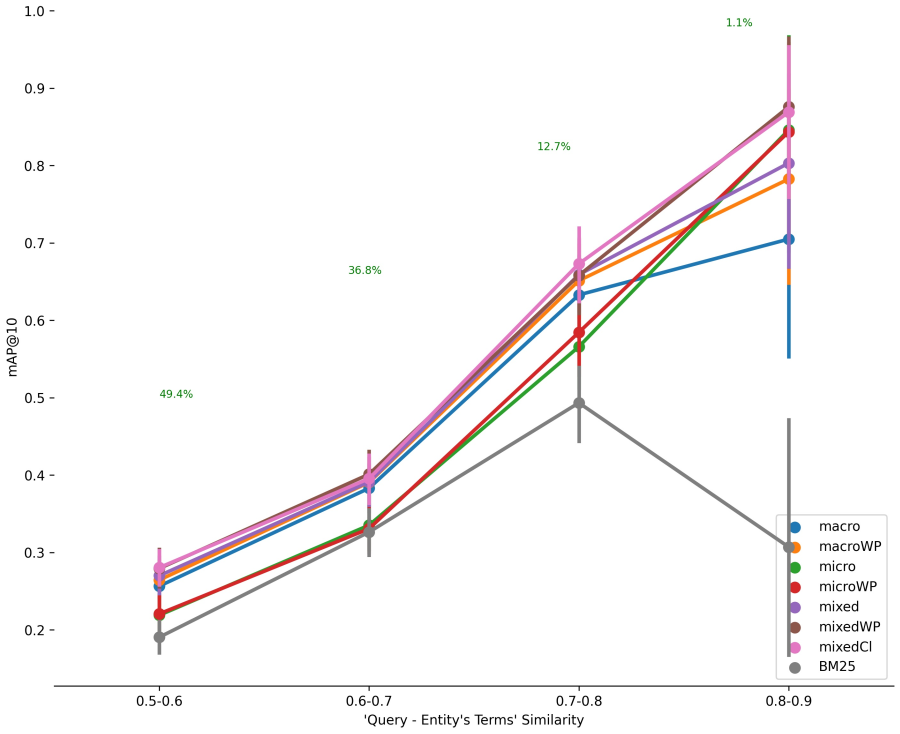

A

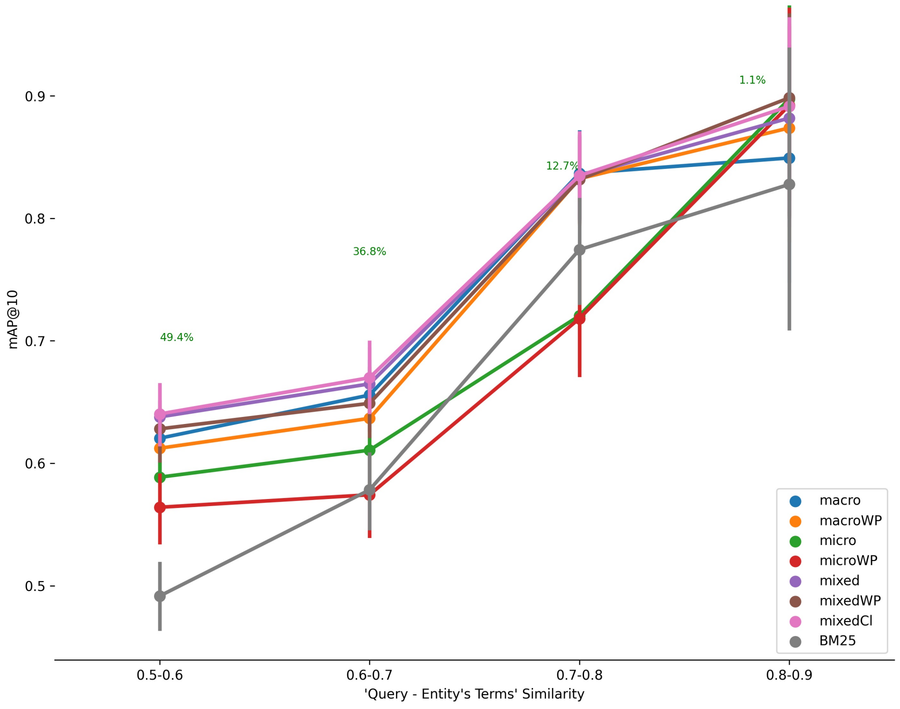

B

Figure S2: The relationship between the similarity of terms in the query with those in the entity to mAP@10 for BioModels-CA dataset. The number of test data is 1,777 and (A) compared to all 54,456 entities and (B) compared to entities 1,538 entities appeared in BioModels-CA
